# Characterization of Genomic Variants Associated with Scout and Recruit Behavioral Castes in Honey Bees Using Whole-Genome Sequencing

**DOI:** 10.1371/journal.pone.0146430

**Published:** 2016-01-19

**Authors:** Bruce R. Southey, Ping Zhu, Morgan K. Carr-Markell, Zhengzheng S. Liang, Amro Zayed, Ruiqiang Li, Gene E. Robinson, Sandra L. Rodriguez-Zas

**Affiliations:** 1 Department of Animal Sciences, University of Illinois Urbana-Champaign, Urbana, Illinois, United States of America; 2 Biodynamic Optical Imaging Center, College of Life Sciences, and Peking-Tsinghua Center for Life Sciences, Peking University, Beijing, China; 3 School of Integrative Biology, Ecology, Evolution, and Conservation Biology Program, University of Illinois Urbana-Champaign, Urbana, Illinois, United States of America; 4 School of Molecular and Cell Biology and Neuroscience Program, University of Illinois Urbana-Champaign, Urbana, Illinois, United States of America; 5 Department of Biology, York University, Toronto, Ontario, Canada; 6 Novogene Bioinformatics Institute, Beijing, China and Biodynamic Optical Imaging Center, Peking-Tsinghua Center for Life Sciences and School of Life Sciences, Peking University, Beijing, China; 7 Carle Woese Institute for Genomic Biology, Department of Entomology, and Neuroscience Program, University of Illinois at Urbana-Champaign, Urbana, Illinois, United States of America; 8 Department of Animal Sciences, Department of Statistics, Neuroscience Program, and Carle Woese Institute for Genomic Biology, University of Illinois at Urbana-Champaign, Urbana, Illinois, United States of America; Universidade de São Paulo, Faculdade de Filosofia Ciências e Letras de Ribeirão Preto, BRAZIL

## Abstract

Among forager honey bees, scouts seek new resources and return to the colony, enlisting recruits to collect these resources. Differentially expressed genes between these behaviors and genetic variability in scouting phenotypes have been reported. Whole-genome sequencing of 44 *Apis mellifera* scouts and recruits was undertaken to detect variants and further understand the genetic architecture underlying the behavioral differences between scouts and recruits. The median coverage depth in recruits and scouts was 10.01 and 10.7 X, respectively. Representation of bacterial species among the unmapped reads reflected a more diverse microbiome in scouts than recruits. Overall, 1,412,705 polymorphic positions were analyzed for associations with scouting behavior, and 212 significant (p-value < 0.0001) associations with scouting corresponding to 137 positions were detected. Most frequent putative transcription factor binding sites proximal to significant variants included Broad-complex 4, Broad-complex 1, Hunchback, and CF2-II. Three variants associated with scouting were located within coding regions of ncRNAs including one codon change (LOC102653644) and 2 frameshift indels (LOC102654879 and LOC102655256). Significant variants were also identified on the 5’UTR of membrin, and 3’UTRs of laccase 2 and diacylglycerol kinase theta. The 60 significant variants located within introns corresponded to 39 genes and most of these positions were > 1000 bp apart from each other. A number of these variants were mapped to ncRNA LOC100578102, solute carrier family 12 member 6-like gene, and LOC100576965 (meprin and TRAF-C homology domain containing gene). Functional categories represented among the genes corresponding to significant variants included: neuronal function, exoskeleton, immune response, salivary gland development, and enzymatic food processing. These categories offer a glimpse into the molecular support to the behaviors of scouts and recruits. The level of association between genomic variants and scouting behavior observed in this study may be linked to the honey bee’s genomic plasticity and fluidity of transition between castes.

## Introduction

Division of labor in honey bee (*Apis mellifera*) colonies is characterized by bees exhibiting distinct behaviors and performing specialized tasks. Among the behavioral castes in a colony, forager bees identify, collect and bring back to the colony pollen, nectar and water. Among foragers, scout bees actively seek new resources and return to the colony to communicate their type, quantity and location to recruit bees that are enlisted to collect these nutritional resources via dance language [1]. Between 5% and 35% of the foragers in a colony display scout behaviors, depending on the colony needs [2,3].

Behavioral castes associated with division of labor show differences in brain gene expression, DNA methylation, non-coding RNA (ncRNA), and neuropeptides [1,4–11]. A microarray study of the expression of 13K transcripts reported that 16% of the genes represented on the microarray were differentially expressed between the brains of scouts and recruits [1]. A number of these genes were associated with the dopamine, octopamine, glutamate, and γ-aminobutyric acid or GABA signaling pathways that have been linked to novelty-seeking and reward-dependent behaviors across behavioral castes [1].

Variation in foraging behaviors has a heritable component [12–14]. Genetic variation has been reported on: the tendency of honey bees to scout [15–18] time of day preference for pollen collection [19]; preference and quantity of collection of pollen and nectar [18,20]; and dance participation [18] in foragers. Honey bee lines selected for high and low pollen hoarding showed significant line differences for foraging-related tasks such as dance performance, scouting, and dance attending [21]. Also, a number of quantitative trait loci (QTLs) in candidate genes associated with the insulin/insulin-like signaling pathway have been associated with foraging behavior [21].

The sequencing of the *A*. *mellifera* genome [22] combined with advances in whole-genome sequencing technologies provide new opportunities to uncover genetic variations associated with behavioral differences. In particular, genome sequencing by synthesis and alignment of the resulting short sequence fragments or reads to a reference genome can be used to identify inter- and intra- behavioral caste genetic variation. The enhanced information resulting from the simultaneous mapping of reads from multiple individuals to the reference augments the accuracy of the mapping and variant calling including single nucleotide polymorphisms (SNPs) and insertions or deletions (indels). The consideration of multiple individual genomes also enhances the characterization of the genomic variant into homozygous and heterozygous genotypes relative to the reference genome sequence.

Genomic variant analysis in the honey bee is further strengthened by the mating and sex-determination manners of this species. The honey bee exhibits polyandry and haplodiploidy that can be exploited to augment the information available and, thus, the precision of whole genome sequencing association studies (WGSAS). Polyandry describes the initiation of a colony by the mating of a single female queen to potentially multiple male drones. Haplodiploidy describes the condition where drones originate from unfertilized eggs and thus are haploid, meanwhile queens and workers (nurses and foragers) are diploid. Exploitation of haplodiploidy occurs simply by mating a single queen exclusively to a single drone. The resulting progeny resembles an inbred backcross scenario for loci where the queen is heterozygous because the drone can only pass one set of alleles in a manner comparable to a homozygous diploid parent. Progeny from a heterozygous queen will therefore exhibit different phenotypes (e.g. scout or recruit) depending on the allele inherited from the queen. When the queen is homozygous for a locus, then all the progeny will have the same genotype with identical phenotype and are thus, indistinguishable.

The overall goal of this study was to identify and characterize genome variants associated with behavioral differences between scouts and recruits. Supporting objectives were to annotate the type of variation (e.g. single nucleotide polymorphism, insertion or deletion), to infer the potential impact of the variation at the transcriptomic and proteomic levels. These objectives were accomplished via a bioinformatics pipeline applied to the analysis of whole-genome sequences of individual bees. Evaluation of unmapped sequence reads offered insights into the reference genome and microbiome of the honey bee.

## Materials and Methods

### Experimental design and sample collection

Scouts and recruits were collected from a small nucleus one-patriline colony at the University of Illinois at Urbana-Champaign. The size of the colony was estimated at 8,000 bees and the bees were collected over a 14 day period in early September 2011. This one-patriline colony derived from a single queen that was inseminated with the semen of a single drone. For the complete experimental period including colony formation, the colony only had a single queen as determined from at least weekly checks. Discrimination between scouts and recruits was accomplished using a modified version of the proven novel-feeder assay [1]. Results from this assay have been confirmed by an independent hive moving assay [1]. Briefly, the colony was placed within an outdoor 6 m x 20 m x 3 m screened enclosure to control the food sources available to foragers. Foragers were allowed to visit a training feeder filled with 50% w/v sucrose and paired with a color and a shape cue. These foragers were marked with Testor’s enamel paint at the feeder. The percentage of foragers among workers was estimated at 25–30%. This percentage is consistent with reports between 5% and 35% depending on forage availability [2,3]. After several hundred foragers were marked, a novel feeder was placed 6 m away from the training feeder. This feeder was paired with a different color and shape cue as well as a novel odor. Foragers that abandoned the training feeder and discovered the novel feeder were identified as scouts and caged. Immediately following each scout collection, a forager visiting the training feeder was identified as a recruit and caged. Both the training feeder and novel feeder were present between 9:00–15:00 hours. At 15:00 hours, all scouts and recruits were frozen with liquid nitrogen and stored at -80°C.

The proportion of scouts relative to recruits, much like the proportion of foragers relative to nurses, and the proportion of other behavior groups in the colony, depends on the colony requirements. Thus, the phenotype studied and the underlying genetics is characterized by predilection and behavioral bias, rather than determinism. Furthermore, the experimental protocol implemented identifies those foragers with a predilection and behavioral bias because the colony had stable requirements and only bees that clearly demonstrated scouting or recruiting behaviors were sampled.

#### DNA extraction

Bees were frozen with liquid nitrogen and the whole body was ground using a Biopulverizer (Biospec Products). Genomic DNA was extracted and purified from these foragers using a Puregene Tissue Kit (Gentra Systems), following the manufacturer’s instructions modified for 20–120 mg whole bees. The pulverized tissue was transferred to 1.5 ml Safe-Lock Tubes (Eppendorf International) and 1200 μl Cell Lysis Solution was added. Each sample was mixed with 6 μl Proteinase K Solution (20 mg/ml) by gentle vortexing and incubated in a shaking water bath at 55°C for at least 6 hours. Another 6 μl Proteinase K Solution was added and mixed by inverting 5 times. The solution was incubated at 37°C for 15 minutes. The sample was cooled to room temperature, and 400 μl Protein Precipitation Solution cell lysate was added. This mixture was vortexed vigorously at high speed for 10 seconds to mix the Protein Precipitation Solution (Qiagen 158912) and incubated at 4°C overnight. The sample was then centrifuged at 13,000–16,000 x g for 10 minutes at 4°C. A pipette was used to move 750 μl of the supernatant into of two 2.0 ml microfuge tubes containing 600 μl 100% Isopropanol, and 1 μl glycogen was added. The sample was mixed by inverting gently 100 times. The mixture was incubated at -20°C overnight. After centrifuging at 13,000–16,000 x g for 3 minutes, the supernatant was poured off, and 600 μl 70% Ethanol was added. This mixture was vortexed until the pellet came loose, and then centrifuge at 13,000–16,000 x g for 3 minutes. The ethanol was poured off, and the pellet was allowed to air dry for 15 minutes at 37°C. After adding 55 μl TE buffer, the DNA was allowed to rehydrate overnight at room temperature on shaker (100RPM) and then stored at -80°C.

### Whole-genome sequencing and read mapping

Whole-genome sequencing was favored over a SNP chip because the former platform captures more classes of variations (e.g. repeats, indels), offers a single base resolution of the entire genome, is not biased or restricted to selected SNPs on chips, and can detect all variations present in individual genomes. Sequencing libraries were constructed using Illumina TruSeq SBS kit following the manufacturer's guidelines (Illumina, CA, USA). Paired-end 100-nucleotide read sequencing was performed using the Illumina HiSeq2000 platform (Illumina, CA, USA). The resulting reads were subjected to quality control using a custom script to remove or trim individual reads of questionable quality or reliability. Pairs of reads were removed when at least one read had low quality or contained an adaptor sequence. Low quality reads were defined as having ≥ 10% unidentified nucleotides or > 50% bases having Phred quality score < 5. A read containing an adaptor sequence was defined as one read with > 10 nucleotides aligned to the adapter and ≤ 10% mismatched nucleotides. Up to 6 terminal nucleotides at the 3’ end of reads were trimmed meanwhile the other member of the read pair was not trimmed in 40 samples (Phred score < 5).

After quality control, the reads were mapped to the *A*. *mellifera* genome assembly version 4.5 (Amel_4.5 GCF_000002195.4) that encompasses 5645 scaffolds including 16 linkage groups (LG or chromosomes) and the remaining scaffolds are unplaced. Bowtie2 version 2.1 [23] with the very-sensitive-local read option was used to map the reads. This enabled further trimming of reads to maximize the alignment score and was used to address possible genetic differences between the current population and the sequenced reference genome.

### Unmapped reads

Homology searches were performed for each sequence that could not be mapped to the reference genome using BLASTN version 2.2.30+ [24] against the NCBI nucleotide collection and taxonomic databases (Dec 2014) [25]. To ensure accurate matches, only matches with e-value < 10^−10^ using default BLASTN settings were further analyzed. The highest scoring match to each read was recorded and matches were summarized across a number of informative taxonomic classifications. Matches to bacterial species were subdivided into the most frequent species. In addition, matches to *Apis* and non-*Apis* Arthropoda species were further sub-classified as: i) repetitive (when the sequence was annotated as repeat, or microsatellite; ii) ribosomal (when the sequence was annotated as ribosomal such as 18S and 28S ribosomal RNA); and iii) “other” for all remaining sequences.

### SNPs calling and association to behavior

SNPs were assigned in each honey bee relative to the reference genome using the *mpileup* option of Samtools version 0.1.19 [26]. Reliable genotyping was based on the consideration of a maximum depth of 100 reads and default settings. Association between SNP allele and genotype frequencies and scout and recruit groups was assessed using a conservative two-tailed Fisher's exact test [27]. For the reference (R) and non-reference (N) alleles, complementary association tests were evaluated including: 1) Genotypic (RR vs RN vs NN); 2) Allelic (R vs N); 3) Dominant gene action of reference allele (RR or RN vs NN); 4) Recessive gene action of reference allele (RR vs RN or NN); and 5) Cochran-Armitage trend that assumes a linear relationship between allele number and behavior. One scout was removed from all association tests due to the limited number of reads. False discovery rate (FDR) [28] adjustment of the test statistic p-value for multiple testing was considered to control for false positive association across the more than 1 million tests. A raw unadjusted p-value < 0.0001 was comparable to a FDR-adjusted p-value < 0.1 and this threshold was used to identify significant associations with scouting behavior.

The genotypic correlations between variants associated with scouting behavior within LG were investigated and visualized using heatmap plots. The genotypes were correlated between positions with behavior group to uncover potential caste-specific disequilibrium. Genotypes were coded 0 (RR), 1 (RN), and 2 (NN) and a linear Pearson correlation estimate was used to summarize the covariation between positions.

The relative location and impact of each SNP on known honey bee genes was determined using the SNPeff tool build 2014-05-01 [29] and the NCBI *A*. *mellifera* genome annotation version 102. Transcription factor binding sites were predicted in genome regions encompassing a variant significant for at least one test using ConSite [30]. A ± 1000 bp region (or the maximum sequence available) surrounding the variant was extracted from the genomic sequence and evaluated for potential transcription factor binding sites using all transcription factor profiles from the insect taxonomic super-group and default settings. Putative transcription factor binding sites with at least an 80% nucleotide identity to a transcription factor profile within the window containing a SNP site were recorded.

## Results and Discussion

### Read mapping

The total number of mapped bases in recruits and scouts was 66.81 and 62.62 Gigabases, respectively. The total coverage depth in recruits and scouts was 245.48X and 235.57X, respectively and the median coverage depth in recruits and scouts was 10.01 and 10.7 X, respectively. The depth of this coverage is higher than the 634X whole genome coverage across 140 bees from 14 populations reported in a comparable study [31]. Reads from at least one honey bee were mapped to 5295 out of 5320 LGs and unplaced scaffold sequences in the reference genome version 4.5. A total of 4595 scaffold sequences (87%) including all 16 LGs had reads from all 22 scouts and 22 recruits evaluated in this study. The coverage of honey bee assembly scaffolds achieved by this study supersedes other whole-genome honey bee sequence studies reporting assemblies of 2,430 scaffolds [32]. The median number of scout reads and reads mapped to the reference genome were 40.2 million and 35.8 million, respectively (S1 Table). The median number of recruit reads and reads mapped were 37.3 million and 33.1 million, respectively. The percentage of read pairs mapped concordantly among all read pairs based on median counts was approximately 86% and consistent between scout and recruit behavior groups. The percentage of read pairs mapped discordantly (pair mates were not in the expected relative orientation, or were not mapped within the expected distance range) among all read pairs based on median counts in recruits and scouts was comparable and approximately 0.76% and 0.52%, respectively. Correctly paired reads, duplicate reads, singleton reads and reads mapped to different recruits were similar between groups, albeit slightly higher in scouts than recruits. The previous metrics confirmed that the selected sequencing approach including 100 nucleotide-long, paired-end reads offered higher mapping depth and accuracy on genotyping and variant calling than alternative setting used in other honey bee sequencing studies [32].

The median percentage of mapped reads within behavior group was approximately 10% higher and less variable in recruits than scouts. The median percentage of mapped reads from recruits was 91.8% and ranged from 85.5% to 96.5% and the median percentage of mapped reads from scouts was 81.6% and ranged from 60.8% to 95.4%. Scouts had more reads per bee than recruits with the median difference of 2.96 million reads. However, this difference was mainly attributed to the higher number of unmapped reads in scouts than recruits with the median difference of 4.6 million reads per bee. Remaining mapping statistics such as proportion of properly paired reads, singleton reads and read mates mated to different LGs were similar between groups and favored scouts.

The median unmapped reads was 2.7 times higher in scouts than recruits. This difference between scouts and recruits in unmapped reads did not influence the whole genome sequencing association study because only mapped reads were used for this analysis. Subsequent study of the unmapped reads offered insights about this difference between behavior groups.

### Study of unmapped sequenced reads

Unmapped reads were subjected to further homology searches to understand the differences in mapping rates between scouts and recruits. Among all unmapped reads, 30% within each group were identified using BLASTN at e-value < 10^−10^ (Table 1). The unidentified sequences among the unmapped reads could be attributed to sequencing-related errors and limited quality resulting in partial matches to known sequences in honey bees or other organisms or limited coverage in the reference database used. Whole-body DNA was sequenced in this study and thus, sequences from the honey bee microbiome were anticipated and detected as a result of our analysis of unmapped reads.

**Table 1:** BLAST Homology Matches of Unmapped Scout and Recruit Sequence Reads by Taxonomic Group.

Taxonomic group^a^		Recruit reads		Scout reads		Total mapped reads		
Kingdom	Rank	Count	%^b^	Count	%^c^	Count	% Recruits^d^	% Scouts^e^
Eukaryota	*Apis*–repeat	431411	0.66	445544	0.69	876955	49.2	50.8
	*Apis*–ribosomal	896949	1.38	959250	1.48	1856199	48.3	51.7
	*Apis*–other	478225	0.74	443472	0.68	921697	51.9	48.1
	Arthropoda- repeat	465	0.00	462	0.00	927	50.2	49.8
	Arthropoda–ribosomal	766865	1.18	828812	1.28	1595677	48.1	51.9
	Arthropoda—other	28727	0.04	59427	0.09	88154	32.6	67.4
	*Nosema*	1277165	1.97	1424226	2.19	2701391	47.3	52.7
	Other Eukaryota	689079	1.06	1031932	1.59	1721011	40.0	60.0
Bacteria	*Bartonella*	151884	0.23	313601	0.48	465485	32.6	67.4
	*Bifidobacterium*	678905	1.05	3483258	5.36	4162163	16.3	83.7
	*Gilliamella*	2254533	3.47	12546569	19.31	14801102	15.2	84.8
	*Lactobacillus*	316338	0.49	2693381	4.15	3009719	10.5	89.5
	*Snodgrassella*	8595784	13.23	20063286	30.88	28659070	30.0	70.0
	Other Bacteria	1053110	1.62	3015850	4.64	4068960	25.9	74.1
Other	Other species	8552	0.01	25439	0.04	33991	25.2	74.8
Total		17627992	27.14	47334509	72.86	64962501	27.1	72.9

^a^ Matches were grouped by kingdom and individual species or phylum depending on overall frequency of matches.

^b^ Percentage of unmapped recruit reads matched by homology searches

^c^ Percentage of unmapped scout reads matched by homology searches

^d^ Percentage of unmapped recruit reads matched to a taxonomic group out of all unmapped reads matched to that taxonomic group

^e^ Percentage of unmapped scout reads matched to a taxonomic group out of all unmapped reads matched to that taxonomic group

#### Taxonomic group matches

Among the unmapped reads that were matched to sequences in the NCBI databases, most matches were to sequences pertaining to the bacteria (85%) and eukaryota (15%) kingdoms with the matches from the other kingdoms amounting to less than 0.5% of all matched reads. Among the species with matched reads, the two most frequent bacterial species were *Snodgrassella alvi* wkB2 and *Gilliamella apicola* that accounted for 42% and 22% of all matched reads, respectively. The most frequent bacterial genuses matched among the unmapped sequences were: *Snodgrassella* (44% of all matched reads), *Gilliamella* (23% of all matched reads), *Bifidobacterium* (6% of all matched reads), *Lactobacillus* (5% of all matched reads), and *Bartonella* (1% of all matched reads). Among the unmapped reads, 4% of these sequences were matched to the unicellular microsporidian bee parasite *Nosema* sp., with *Nosema ceranae* BRL01 accounting for 3.9% of the matched reads. Approximately 5.5% of the unmapped reads matched to Apis species and *A*. *mellifera* (including subspecies), *Apis florea*, and *Apis dorsata* accounted for 3.1%, 1.4%, and 1.0% of these reads, respectively. The Hymenoptera order excluding *Apis* species and other insects excluding the Hymenoptera order accounted for 2% and 1% of all matched reads, respectively. Only 0.1% of the reads were matched to other non-insect species in the Arthropoda order. Other notable matches that accounted for 2.7% of all unmapped reads included vertebrate (1.25%), plants (0.42%), fungi (0.04%), and other Metazoa species (0.21%). These matches to unmapped reads could be attributed to the whole body DNA extraction protocol, contamination and sequencing errors [33–35].

A component of the higher percentage of unmapped reads in scouts relative to recruits corresponded to a higher number of matches to bacteria species. Within bacteria grouping, 67 to 89% of the reads corresponded to scouts reads. When analyzed as a proportion of the total number of reads in scouts and recruits, recruits had 6.4% more reads from *Snodgrassella* than scouts (48.8% of recruit reads minus 43.4% of scout reads). However scouts had 13.7%, 3.9% and 3.5% more reads from *Gilliamella*, *Lactobacillus*, and *Bifidobacterium* species, respectively, than recruits. Unmapped reads that matched to *Bartonella* and other bacterial species exhibited < 0.5% difference between behavior groups.

The consistent differences in bacterial annotation among unmapped reads between the 44 sequenced genomes across behavior groups reflects real differences between scouts and recruits due to the similar collection protocols, occurrence in multiple individuals, large library size, and the wide variations between different bacterial species. Our findings confirmed prior reports of predominance of *Snodgrassella*, *Gilliamella*, *Lactobacillus* and *Bifidobacterim* species among gut microbiote in honey bee foragers and adds to reports of microbiome differences between castes [36–39].

Differences between behavior groups for some but not all bacteria species identified in our study reflect the more diverse microbiome of scouts relative to recruits. These differences are likely due to scouts visiting more locations resulting in a more diverse bacterial population than recruits. Evaluation of the prevalence of individual bacterial species based on our sequencing study should be undertaken with caution because the frequency of bacterial accession numbers is expected to be biased towards genomic sequences rather than specific marker genes (e.g. 16S ribosomal RNA gene). This trend is linked to the randomly primed and sequenced DNA segments obtained from the sequencing platform used and the availability of whole bacterial genomes in the nucleotide database used for BLASTN matches. Nevertheless, the consistency in bacterial detection across the 22 scouts and 22 recruits in this study and with previous independent studies suggests that bacterial sequences reads can offer further evidence of differences on microbial exposure and sustainment between scouts and recruits.

*Apis non-mellifera matches*. Approximately 3% of the unmapped reads within scouts and recruits matched to Apis sequences that were classified as either ribsomal (50%), repetitive (24%) or other (26%). In addition, 1% of the unmapped reads were matched other Arthropoda sequences and the majority of these (95%) were ribsomal sequences.

Unmapped sequences that were matched to *Apis* species other than *A*. *mellifera* (Apis non-mellifera) exhibited similar distribution between scouts and recruits. The median number of reads per gene and honey bee that matched to other *Apis* non-mellifera genes ranged from 3 to 15. Evidence of this included the low variability of the number of *Apis* non-mellifera matched reads across individual bees regardless of behavior group. Also, a substantial number of unmapped reads exhibited matches to *Apis* non-mellifera annotated regions that, in turn, exhibited partial homology to the *A*. *mellifera* genome. Three of the most frequent *Apis* non-mellifera read matches that accounted for 0.94% of the unmapped reads corresponded to *A*. *dorsata* genes (titin-like, histone-lysine N-methyltransferase 2C-like, and histone acetyltransferase KAT6B-like). These three genes were in turn partially matched to the *A*. *mellifera* genome version 4.5. Two *A*. *dorsata* genes, uncharacterized LOC102671240 and mucin-2-like, matched to 0.36% and 0.26% of the unmapped reads, respectively, had no matches to the *A*. *mellifera* genome version 4.5 but had evidence that these genes also are present in the *A*. *mellifera* genome. Unmapped reads also matched two rRNA A. *florea* genes that were in turn matched to partial *A*. *mellifera* rRNA genes. Moreover, reads were matched to an *Apis* non-mellifera Ava I repeat and a microsatellite 4A46 sequences however these segments are not present in the *A*. *mellifera* genome version 4.5. These results suggest that the unmapped reads were likely due to limited coverage of some regions the *A*. *mellifera* genome version 4.5 as well as sequence quality, rather than actual differences between scouts and recruits.

#### Uninformative *A*. *mellifera* unplaced scaffolds

Homology searches of all unplaced scaffolds in the *A*. *mellifera* genome version 4.5 further the understanding of the difference in unmapped reads between scouts and recruits. A total of 119 unplaced scaffolds only had BLASTN matches to bacterial sequences (e-value < 10^−10^) in the NCBI nucleotide database. These scaffolds were removed from the association analysis because variants in these scaffolds are likely to be unrelated to causal differences between behavior groups.

### Allele and genotype calling

Using the *A*. *mellifera* genome version 4.5 as the reference genome, 2,228,909 positions exhibiting 2 alleles were identified across the scout and recruit genomes studied after filtering for the 199 unplaced scaffold of apparent bacterial origin and one atypical scout with limited (< 3X) genome coverage. Of these, 589,098 positions (26% of all positions) exhibited no variation between honey bees and were completely homozygous for the N allele indicating the reference genome was not present in the parents used. In addition, 816,204 positions exhibited no variation between honey bees and were completely heterozygous, indicating that all bees inherited different alleles from each parent. Therefore, the remaining 1,412,705 positions exhibiting allelic variation between honey bees were tested for associations with scouting behavior by comparing the distribution of alleles and genotypes among scouts and recruits (Table 2).

**Table 2:** Number of Variants by Association Test Result and Location.

Association test significance^a^				Variant location								
Genotype	Trend	Allelic	R/N	Coding	Intron	3’UTR	5’UTR	Down-stream	Up-stream	Inter-genic	Other	Total
NA^b^	NA	NA	NA	10899	373185	7995	3266	31501	32049	119824	10379	589098
NS	NS	NS	NS	28059	1028376	21149	8141	83270	87751	348414	34514	1639674
NS	NS	NS	S	0	1	0	0	0	0	0	0	1
NS	NS	S	NS	1	16	1	0	1	2	15	0	36
NS	S	NS	NS	1	4	1	0	3	0	7	0	16
NS	S	S	NS	0	0	0	0	0	0	1	0	1
S	NS	NS	NS	1	7	0	0	0	3	13	0	24
S	NS	NS	S	0	1	0	0	1	0	0	0	2
S	S	NS	NS	0	24	0	1	3	0	17	0	45
S	S	NS	S	0	5	0	0	0	1	3	0	9
S	S	S	S	0	2	0	0	0	0	1	0	3
Significant for at least one test				3	60	2	1	8	6	57	0	137
Non-Significant for any test				38958	1401561	29144	11407	114771	119800	468238	44893	2228772
Total number of Variants				38961	1401621	29146	11408	114779	119806	468295	44893	2228909

^a^ Geno: Genotypic test (RR vs RN vs NN genotypes where R is the reference genome allele and N is the non-reference genome allele); Trend: Cochran-Armitage trend test; Allelic: allelic association (R vs N): Ref/N Dominant gene action test of the reference and/or non-reference allele.

^b^ S: significant where the test(s) p-value < 0.0001; NS: non-significant where the test(s) p-value > = 0.0001; NA: test could not be performed due to limited or lack of genotypic or allelic variation such as when all bees were homozygous for one allele.

Genomic variants associated with scout and recruit behavior were identified in a colony derived from one queen and one drone. The benefit from this shared founder structure is a single stratum that is expected to increase the power to detect real associations over other experimental designs by reducing the ‘background noise’ caused by between individual variants unassociated with behavioral differences. This reduced noise stems from that the coefficient of relationship is typically higher under haplodiploidy than other sex determination systems. For example, the probability that full sisters (same parents) would inherit the same allele is 75% under haplodiploidy but only 50% under the more common XX/XY sex-determination system. However, this population structure could also increase the risk to miss variants associated with behavior. Nevertheless, the population structure and the haplodiploid sex-determination system in honey bees enabled the identification of more than 1.4 million polymorphic positions across the genome. The level of polymorphism detected in this population could be related to reports of higher crossover rate in honey bees relative to other species [40–42]. Prior studies have suggested that selection pressure may be at least partially responsible for the capability of the queen to maintain genetic variation [43].

### Whole-genome sequencing association analysis

Genome-wide analysis identified 212 significant (p-value < 0.0001) associations between behavior and alleles or genotypes corresponding to 137 positions (S2 Table). The vast majority of the variants (86 variants) exhibited a significant association between genotype and scouting behavior that was confirmed by the Cochran-Armitage trend test. The most common scenarios were a significant association between scouting behavior and allele (36 positions) or genotype (24 positions) effects. Among the variants with at least one significant association test, the distribution of the genotypes at the significant positions followed the theoretical Hardy-Weinberg expectations under haplodiploidy and sampling variation for the vast majority of the positions. Negligible impact of sequencing errors are expected to have contributed to the genotype distributions on account of the informative SNP calling procedure based on 100X sequence depth that was used. Thus, the one-patriline colony experimental design and the haploidiploidy system in bees are at the core of the genomic similarities between behavior groups.

Most variants associated with scouting behavior were located on assembled LGs (115 variants) and the rest of the variants were distributed on unplaced scaffolds. Most variants on the assembled LGs were located on LG12 (62 positions) including 33 mapped to intergenic regions located 5000 bp from annotated genes and introns. LG5 encompassed the second largest number of significant variants (16 variants) associated with scouting behavior, including 13 variants located within introns. The majority of the variants assigned to unplaced scaffolds (50 out of 56 positions) were classed as intergenic. Among the 137 variants associated with scouting, 10 clusters included at least one pair of variants located < 300,000 bp apart that corresponds to average linkage disequilibrium r^2^ = 0.15 in *A*. *mellifera* [31]. Of these, 7 clusters included 2 positions, 6 clusters were located in intergenic regions, 3 clusters were located in introns, and 1 cluster was located nearby, upstream of a gene. Of the 11,000 predicted genes, the annotation release 102 on the *A*. *mellifera* genome assembly version 4.5 includes approximately 5000 annotated genes such that a number of variants associated with scouting were mapped to uncharacterized genome regions and meagerly characterized genes.

### Transcription factor binding sites proximal to scouting variants

Genome segments matching (> 80% nucleotide identity) transcription factor binding sites were detected within ± 1000 bp from 63 variants associated with scouting behavior. The most frequent binding sites corresponded to the putative transcription factors: Broad-complex 4 (18 positions), Broad-complex 1 (16 positions), Hunchback (13 positions), and chorion factor 2 (CF2-II, 13 positions). There were 11, 14, and 39 positions with 3, 2, and 1 putative transcription factor binding sites, respectively. These overlapping predictions of multiple insect transcription factor binding sites stems from the similarity in the sequence profiles of these transcription factors.

The expression of Broad-complex, Hunchback and CF2-II transcription factors has been reported in previous honey bee studies [44,45]. The association between these transcription factors and scouting behavior can be explained by recent functional studies demonstrating their relationship with motor and behavioral phenotypes [46]. Broad-complex regulates insect salivary gland development and function and metamorphosis and this could be linked to differences between scouts and recruits in the tendency to explore new food sources and the amount of food collected. Also, Broad-complex is a key regulator of the ecdysone cascade and, in turn, ecdysteroides regulate behavioral and physiological events [47]. Hunchback influences the temporal control of neuroblast identity in fruit fly motoneuron lineages [48] and a similar association in honey bees could be linked to differences in motor behavior between scouts and recruits. Hunchback is expressed in the nervous system and has a role on patterning of the central nervous system [46]. The zinc finger protein CF2 is an activator of muscle structural genes in the body wall muscles of fruit fly [49].

### Scouting-associated variant characterization

The locations of the significant variants point to a regulatory capacity consistent with observed differentially expressed genes between scouts and recruits [1]. The location of the variants associated with scouting behavior included: 3 positions in codons, 1 position in a 5' untranslated region (UTR), 2 positions in 3'UTRs, 60 positions in introns, 14 positions nearby genes, and the remaining 57 positions in intergenic locations distant from known annotated genes. Among the variants associated with scouting, 21 positions were indels located in UTRs (3 positions), introns (25 positions), nearby genes (9 positions), and intergenic regions (26 positions). Simultaneous consideration of different combinations of even a few locations is sufficient to provide complete genetic discrimination between behaviors. Recognizing the continuum in behaviors, results and discussion focus on locations where there was distinct differences in the number of scouts and recruits with different genotypes.

#### Variants associated with scouting in codons

The 3 variants in exon or coding regions occurred in 3 long ncRNAs and included one codon change (LOC102653644) and 2 frameshift indels (LOC102654879 and LOC102655256). These ncRNAs have been predicted in *Apis*, *Bombus* and other insect genomes. Long ncRNA could be protein coding or involved in gene regulation. In these cases, characterization of codon change could help understanding the gene function of the actual gene (if the ncRNA is protein coding) or target gene (if the ncRNA is regulatory). The variant (located at position 2160939) within LOC102653644 (located on LG12) resulted in a Guanine to Cytosine transversion. All recruits except one were homozygous for the reference allele for this ncRNA LOC102653644 variant compared to 10 scouts that were homozygous for the reference allele. The remaining recruit and 4 scouts were heterozygous. This distribution resulted in a significant trend test (p-value <0.0001) and slightly less significant genotypic (p-value < 0.0002) and allelic (p-value < 0.0004) tests. This ncRNA is located proximal to LOC410058, a myosin light chain alkali-like gene. Myosins are key proteins in the nervous and visual systems of honey bees [50].

The variant (located at position 1154) within ncRNA LOC102655256 (mapped on unplaced scaffold NW_003382516.1) is characterized by a single Thymine deletion relative to the reference genome. This position was primarily heterozygous in scouts (18 bees) but varied between heterozygous (10 recruits) and homozygous (11 recruits) for non-reference allele. This structure resulted in significant allelic and genotypic tests.

The variant (located at position 8609127) within ncRNA LOC102654879 (located on LG10) was characterized by a deletion of 2 Thymines (TT) compared to the reference genome. The majority of the recruits were homozygous for the reference allele and a majority of scouts were heterozygous. This position is proximal to the membrin gene that also includes a SNP on the 5’UTR region. This location is also located within an intron of LOC412914, an uncharacterized gene that, based on a BLASTN homology search, appears to be similar to a serine/threonine-protein phosphatase 2A regulatory subunit B' subunit alpha.

#### Variants associated with scouting in untranslated regions

A nucleotide transition (Guanine in the reference genome to Adenine) located at position 8624631 on LG10 maps to the 5'UTR of membrin. This position is 46 bp upstream of the membrin initiation codon and 52 bp upstream of beta-catenin-like protein 1-like (Ctnnbl1 or LOC409671) although there is no evidence of an overlap between the 5'UTRs of membrin and Ctnnbl1. For this variant, 20 scouts were homozygous for the reference allele whereas the recruits were almost evenly split between homozygous for the reference allele and heterozygotes. Membrin codes a target membrane ER-Golgi Soluble NSF Attachment Protein Receptor (SNARE) protein that is involved in protein transport. Dysregulation of membrin may disrupt multiple other SNARE proteins that together reduce bacterial replication in fruit fly [51]. Modification on membrin expression may counteract the potential higher risk to pathogen exposure of scouts relative to recruits. Ctnnbl1 codes for a spliceosome-associated protein [52].

A deletion of an Adenine relative to the reference genome (located at position 5500706) in the 3'UTR of laccase 2 (Lac2), located on LG12, was associated with scouting. This variant is located 42 bp after the stop codon and 2706 bp before the end of the mRNA. The majority of the recruits (18 bees) were heterozygous for the Lac2 alleles with 2 recruits homozygous for either Lac2 allele meanwhile two times more scouts were homozygous for the non-reference Lac2 allele than heterozygous. The Lac2 plays a critical role as phenol oxidase in body wall sclerotization, pigmentation and maturation of exoskeleton in honey bee and red flour beetle [53,54]. Differential exoskeleton adaptations including wing development may influence the capability of scouts to endure additional perils of scouting new food source or the capability of recruits to carry more food back to the colony. Scouts had significantly less wing damage than recruits [55] and wing damage has significantly deleterious influence on bee foraging behavior and performance [56,57]. Also, differential expression of Lac2 has been associated to insectide resistance in the mosquito *Culex pipiens pallens* [58] and has been detected in the foregut of the beetle *Callosobruchus maculatus* [59]. The potential ways by which Lac2 can influence scouting tendencies need to be elucidated.

A deletion of a Thymine position from the reference genome in the 3'UTR of diacylglycerol kinase theta-like (Dgkq or LOC408809, located on LG4, position 6472951) was associated with scouting. This variant is located 2406 bp after the stop codon and 1793 bp before the end of the mRNA. All except 4 scouts were homozygous for the non-reference allele and the rest were homozygous for the reference allele. In contrast, the opposite pattern in Dgkq genotypes was observed with the recruits with double the recruits homozygous for the reference allele than those homozygous for the non-reference allele. The correlation of genotypes among positions associated with scouting behavior in LG4 showed a similar pattern. The genotype in position 6472951 was negatively correlated with other LG4 positions (5736174, 5812780, 5816424, and 5823271) associated with scouting in scouts and positively correlated in recruits. Dgkq is a neuronal enzyme that participates in signaling cascades and has a role in brain development [60,61].

#### Variants associated with scouting in introns

The 60 variants associated (p-value < 0.0001) with scouting behavior located within introns mapped to 39 genes. Most of the variants were SNPs (27 transitions and 7 transversions) and remainder (8) were indels. The genes including most variants were: LOC100578102 (6 variants), solute carrier family 12 member 6-like (Slc12a6; 5 variants) and LOC100576965 (5 variants).

The 6 variants located in introns of ncRNA LOC100578102 (located on LG12) were all > 4200 bp apart from each other with exception of two positions (positions 3535855 and 3540115) that were 288 bp apart. Almost all recruits (20 or 21) were homozygous for 5 of the 6 variants in LOC100578102 and the scouts were almost equally distributed between the homozygous and heterozygous genotypes.

The 5 intronic variants mapped to Slc12a6 (located on LG5) included 4 positions within 22 bp with the remaining positions (positions 11062231 and 11063028) more than 6300 bp apart. Slc12a6 codes for K+-Cl–cotransporter 3 (KCC3) that is critical both cell swelling and neuronal hyperexcitability with KCCs playing key role on cell volume maintenance in the nervous system of fruit flies [62]. The genotype distribution of the Slc12a6 variants was unusual in that all three genotypes were observed and that for any variant, the 2 behavior types favored different homozygous genotypes.

The 5 intronic variants mapped to LOC100576965 (an uncharacterized meprin and TRAF-C homology domain containing gene) were located > 9000 bp apart with exception of two variants 800 bp apart from each other (positions 11012508 and 11012538). Another nearby LG5 variant (located at position 11048772) was detected 127 bp upstream of LOC100576965 and 299 bp downstream of bromodomain containing 7 (Brd7). Almost all scouts were homozygous for the non-reference allele at most of these positions; 20 bees for position 11048772 and 17 bees for positions 11012508, 11012538 and 11095696. In contrast, only 5 to 8 recruits were homozygous at these positions. LOC100576965 contains a MATH (meprin and TRAF-C homology) domain and Brd7 is associated with neuron projection morphogenesis processes in the fruit fly. Both of these genes were over expressed in honey bees that display Varroa sensitive hygiene behavior (i.e. detection and removal of brood parasitized by the mite *Varroa destructor*) relative to those that do not suggesting that this gene is associated with collective defense [63]. Brd7 codes a nuclear protein that has inflammation and tumor suppressor roles and inhibits cell growth and cell cycle progression by regulating cell-cycle genes at the transcriptional level [64]. Other intronic variants associated with scouting behavior were mapped to LOC100576700, LOC408602, LOC102656849, LOC408372, LOC724833, LOC102656913, and LOC551490. Two transitions (Cytosine to Thymine) located at positions 2097573 and 2097586 on LG1 within LOC100576700 (neurogenin-3-like gene that is a transcription factor involved in neuronal differentiation), were 13 bp from each other and exhibited identical genotype distributions. Almost all scouts were homozygous for the reference allele compared to 9 recruits. The closest variant associated with scouting on LG1 was located at position 2179980 on LOC408602 (predicted myocardin-related transcription factor A-like). LOC408602 is also a transcription factor and has been associated with respiratory and wing development in fruit fly [65].

The variants in LOC102656849 (ncRNA on unplaced scaffold NW_003378247.1), LOC408372 (poly(rC)-binding protein 3-like on LG12) and LOC724833 (matrix metalloproteinase-25-like on LG1) had all except 1 recruit homozygous for the R allele and 12 scouts heterozygous. One intron position within LOC102656913 (ncRNA on unplaced scaffold NW_003378229.1) and 2 close intron positions within LOC551490 (collagen alpha chain CG42342-like on LG12) had all scouts homozygous for the reference allele and the recruits almost equally split between heterozygotes and homozygotes for the R allele.

#### Variants associated with scouting in regions proximal to genes

Proximal to annotated genes, 14 variants associated with scouting behavior were detected between 146 and 844 bp from 12 annotated genes. These variants were located on different LGs with the exception of two cases. The first case includes 2 variants on LG4 (positions 5823271 and 5816424) were located 1414 bp downstream and 3800 bp upstream of cuticular protein 19 (CPR19). At both positions most scouts were homozygous for the non-reference allele and most recruits were homozygous for the reference allele. This gene is over-expressed in nurse relative to forager honey bees manipulated to exhibit nurse behavior and has been suggested that this is key to upholding cuticular structure [66]. Cuticular proteins are critical in restricting water loss, digestion, moulting, defense/immunity and resistance to pathogens. The second case includes 2 variants on LG16 located 3944 and 3946 bp upstream of LOC100578662 (probable G-protein coupled receptor Mth-like 1-like). Both variants had 21 heterozygous recruits meanwhile scouts were distributed over the possible 3 genotypes. LOC100578662 is over-expressed in foragers relative to winter bees that survive for up to 10 months and is linked to determination of adult life span [66].

Among the variants associated with scouting and located in the proximity of annotated genes, a variant was located on LG6 (position 16625187), 76 bp downstream of Sds22, a regulatory subunit of the type 1 protein phosphatase. The protein coded by this gene has been associated with regulation of epithelial cell shape and polarity [67]. For this variant, 21 recruits but only 12 scouts were homozygous for the reference allele. Also, a variant on LG2 (position 7064150), 188 bp downstream of LOC412780 (transmembrane protein 8A-like), was associated with scouting behavior with double the number of scouts (20 bees) than recruits homozygous for the N allele. This gene includes epidermal growth factor-like (EGF-like) and DUF3522 domains. On LG4 (position 6418141), a variant 1016 bp downstream from LOC100578446 is associated with scouting behavior. More than 3 fold more recruits (21 bees) than scouts were heterozygous at this position meanwhile most other scouts (13 bees) and 1 recruit were homozygous for the N allele. LOC100578446, predicted pituitary homeobox homolog Ptx1-like, contains a homeodomain, DNA binding domains involved in the transcriptional regulation of key eukaryotic developmental processes and an OAR domain. This gene is highly similar to the transcriptional regulator pituitary homeobox1 (Pitx) that targets neuropeptide-encoding genes in the domestic silkworm *Bombyx mori* [68]. Lastly, a variant on LG13 (position 9587246) located 3196 bp downstream of cytochrome P450 6AS3 (CYP6AS3) was associated with scouting behavior. Nearly three times more recruits (20 bees) than scouts were homozygous for the N allele at this position meanwhile most of the other scouts (11 bees) and one recruit were heterozygous. Members of the CYP6AS subfamily are involved in the metabolism of quercetin, a main flavonoid component of honey and pollen [69]. The CYP6AS subfamily enables honey bees to be well-equipped to process phytochemicals common in major food sources (i.e., honey and pollen). Honey bees also forage and process nectar and this has been associated with the expansion of CYP6AS sequences in their genome relative to solitary bees, which consume nectar and not honey and forage from a narrower range of floral sources [69]. These observations about links between food sources, processing and CYP6AS could be linked to our findings of differential variant association with scouting since scouts exhibit exploration and food location communication behaviors and recruits are tasked with focused retrieval of nectar and pollen.

#### Variants associated with scouting in intergenic regions

Most variants associated with scouting behavior within intergenic assignments (> 5000 bp from annotated genes) where located on LG12 (33 positions) or on unplaced scaffolds (17 positions). Most of the variants on LG12 were distant, and only two regions separated more than 4000,000 bp on LG12 encompassed 5 variants within 350 bp and 10 variants within 580 bp, respectively. As expected, positions within these 2 small regions exhibited similar genotype distributions among scouts and recruits. Almost all recruits were homozygous for the R allele in the first region and heterozygous in the second region meanwhile the scouts exhibited all genotypes in both regions. At present the understanding of the role of intergenic locations reflects the current knowledge of honey bee genome assembly and annotation. Although many intergenic variants exhibited highly distinct genotype distribution between scouts and recruits, there is insufficient annotation evidence to directly associate these variants to annotated genes.

### Correlations between variants within scouts and recruits

**Fig 1:**
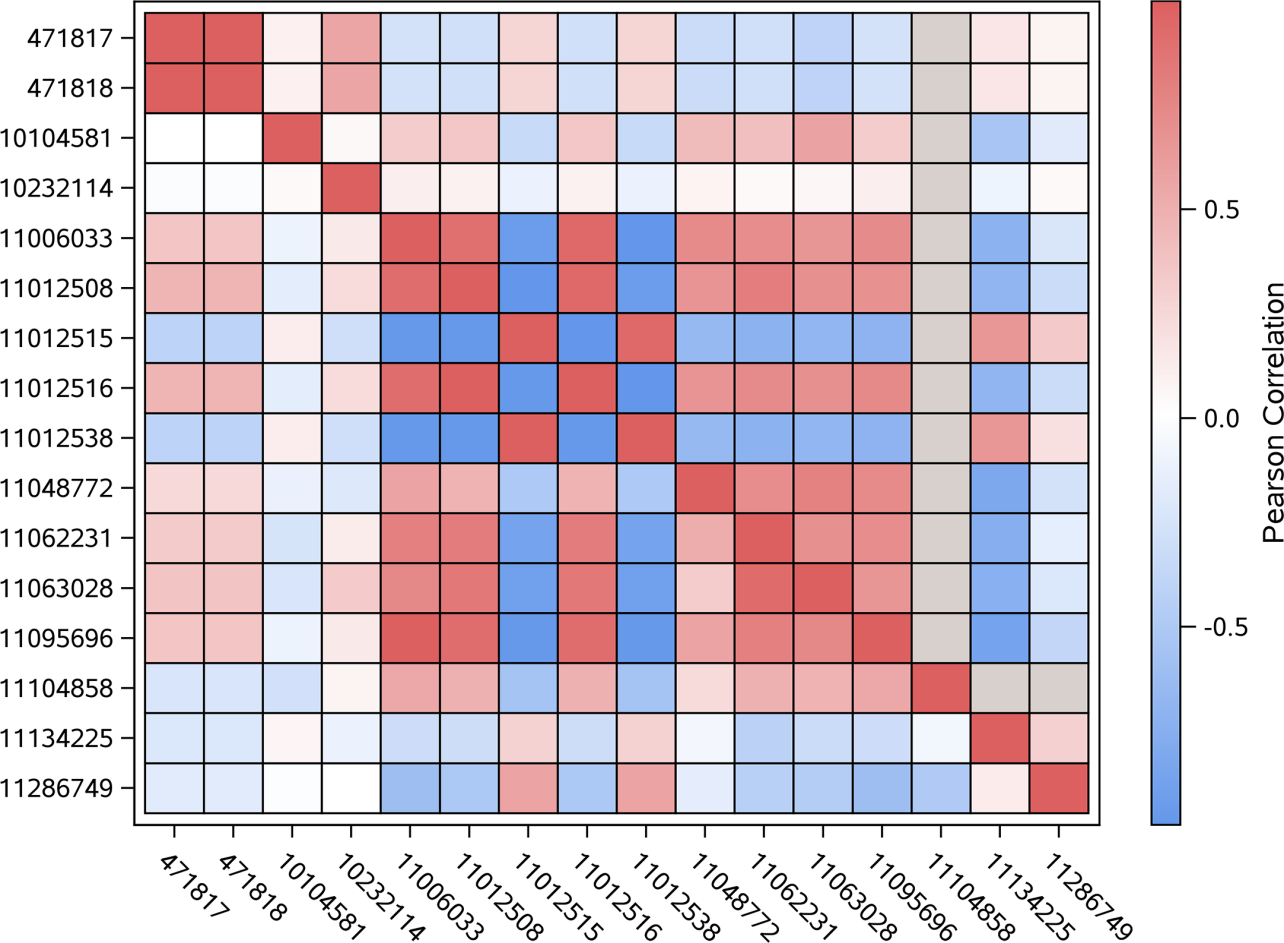
Heat map depicting the correlation between genotypes across variants associated with scouting behavior located on LG5 (NC_007074.3); location in bp is denoted by the rightmost number and gray color indicates no genotypic variation among all bees within one behavior group.

**Fig 2:**
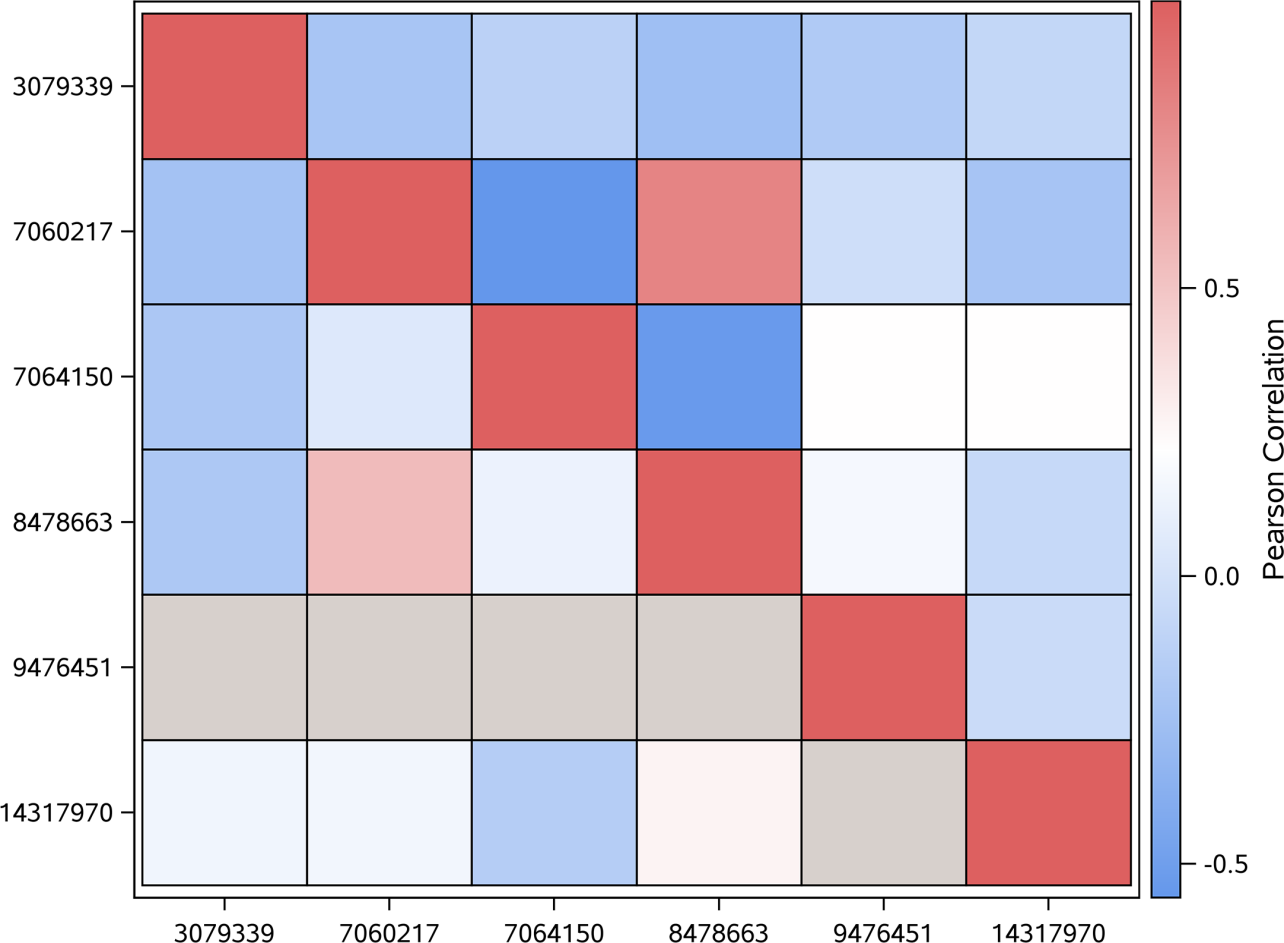
Heat map depicting the linear correlation between genotypes across the variants associated with scouting behavior located on LG2 (NC_007071.3); location in bp is denoted by the rightmost number and gray color indicates no genotypic variation among all bees within one behavior group.

**Fig 3:**
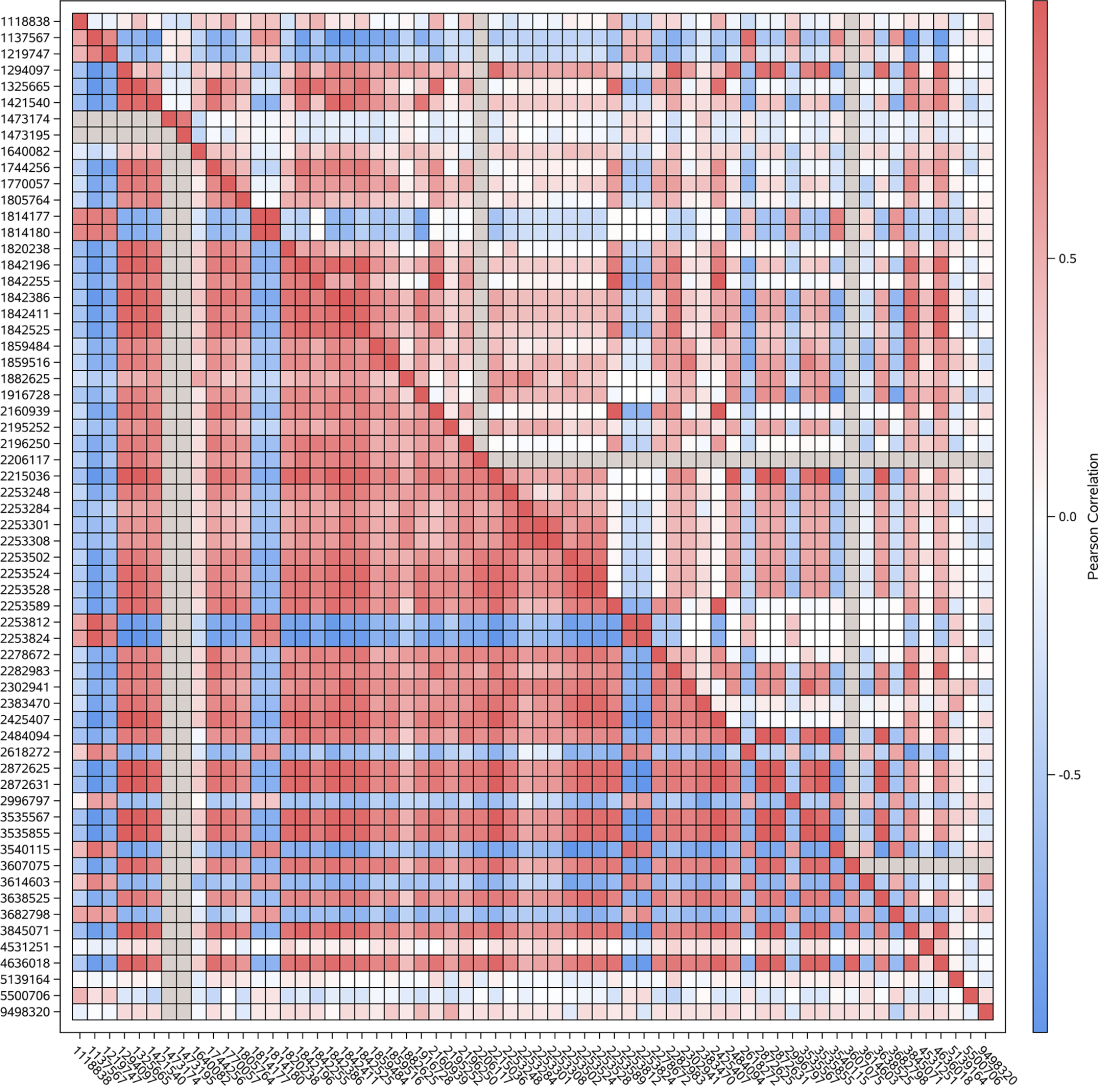
Heat map depicting the linear correlation between genotypes across the variants associated with scouting behavior located on LG12 (NC_007078.1); location in bp is denoted by the rightmost number and gray color indicates no genotypic variation among all bees within one behavior group.

The genotypic correlations between variants associated (p-value < 0.0001) with scouting behavior within linkage group were summarized using heatmap plots (Figs 1, 2 and 3). Positive correlation estimates indicate predominance of equal genotypes (expressed in terms of reference and non-reference alleles between positions), meanwhile negative correlation estimates indicate predominance of distinct genotypes between pairs of variants. Lower (upper) diagonal cells depict the correlations between pairs of variants within scouts (recruits). Grey cells denote lack of genotypic variability (fixed genotype) among honey bees within the behavior caste for the corresponding variant.

The correlations of variants associated with scouting within LG5 depicted in Fig 1 correspond to positions associated with Slc12a6 (positions 11006033 through 11012538) and LOC100576965 (positions 11048772 through 11134225). The highest correlations (> 90%) were observed between the positions located in the intron of Slc12a6 (positions 11006033, 11012508, 11012515, 11012516, 11012538, and to lesser extend 11048772) despite the distance between the first and other 5 variants. The sign and magnitude of the correlations tended to be similar between recruits and scouts although scouts had slightly lower correlations than recruits within LOC100576965.

Most of the correlations between variants associated with scouting behavior within LG2 were negative and tended to be more extreme in recruits (positions 7060217, 7064150, and 8478663) than scouts (Fig 2). Position 7060217 is located in an intron of LOC412780 (transmembrane protein 8A-like), position 7064150 is located 188 bp downstream of LOC412780 and position 8478663 is located in a intergenic region. The predominance of negative correlations may indicate high recombination between the positions studied. Recruits tended to have more extreme correlations than scouts suggesting that variants in these LG2 genes could be beneficial for recruit behaviors. The gray color denoted identical genotypes among all the honey bees in the behavior group and the only fixed position (position 9476451) in LG2 was found among scouts.

Most of the significant variants associated with scouting behavior within LG12 corresponded to 2 large intergenic regions (positions 1805764 through 1859516 and positions 2195252 through 2302941). Ignoring these 2 regions to facilitate visualization, variant genotypes exhibited more extreme correlation among scouts than recruits (Fig 3). This pattern was also observed when all LG12 variants, including those in intergenic regions, were evaluated. The more extreme correlation suggested that those combinations of variants could be associated with scouting behavior. Only the positions on LOC100578102 showed similar variant allele distribution between scouts and recruits. The consecutive fixed positions 1473174 and 1473195 in scouts together with the high to moderate correlation between preceding positions 1118836 to 1421540 and positions 1744256 to 4636018 (and more extreme than in recruits) suggest that this region supports scouting behavior.

### Whole-genome association findings

The level of association between variant and scouting behavior observed in this study could be associated with the honey bee’s known genomic plasticity and fluidity of transition between nurse, recruit and scout behaviors. The number of foragers that show scout behavior is linked to the colony needs and genetic diversity [2,13,18,69]. Indirect evidence for this genetic diversity on behavior is that novice foragers were almost equally divided between those that were recruited by dance signals (recruit behavior), those that pursued searches independent of dance signals (scout behavior) or some combination of recruit and scout behaviors to find their first food source [3]. Further, there was no difference in the number of trips between these groups before finding the first food source although novice foragers tended to use the same approach to find food in successive trips [3]. Genetic differences are also a possible explanation for the differences between scout and recruits for reversal-learning abilities for odor [55]. A multifaceted composition among scouts has been proposed. Instead of one monolithic scout caste, the modified concept distinguishes three types of foragers that may be involved in the exploration behavior of the colony: novice honey bees that become scouts; unemployed experienced honey bees that scout, and lost recruits (honey bees that discover a food source other than the one to which they were directed to by their nest mates [69]. This continuum in behaviors hinders the penetrance of variants associated with scouting behavior, reducing the signal:noise ratio and reducing the proportion of individuals with the causative variant that exhibit scout or recruit behaviors. Additional sequencing studies (whole genome or targeted sequencing of auspicious regions such as those identified in this study on LG10, LG5, and LG12 (including ncRNA and partially annotated genes), will narrow down potentially causative mutations.

A number of functional categories were identified among the genes linked to variants associated with scouting. These categories were associated with neuronal function including: motoneurons, neuroblasts, neuronal enzymes, neuronal projections and neuropeptides. Also, categories related to exoskeleton, epidermal and cuticular development and maturation, immune response, salivary gland development, and enzymes involved in processing food were identified. These categories suggest that the corresponding mutations could be causal, enabler or supportive of the role of scouts exploring a wide range of habitats to uncover new food sources and communication of the location of the sources or of the role of recruits to effectively retrieve food in sufficient amount to ensure the subsistence of the colony.

## Conclusions

Whole-body DNA sequencing of 22 scouts and 22 recruits enabled the uncovering of molecular differences between the behavior groups identified from the reads both unmapped and mapped to the genome. Novel findings from the study of unmapped reads included the higher number unmatched reads, higher match to bacterial sequences, and higher diversity of bacterial sequences in scouts relative to recruits. The most common bacterial taxonomic units in both behavior groups were consistent with prior microbiome studies of forager honey bees. The higher variability among the most frequent bacteria species identified in scouts could be related to the wider-range of habitats visited by scouts while searching for new food sources compared to recruits that concentrate on the fewer and more abundant sources communicated to them by the scouts.

The similar coverage depth between the scout and recruit genome sequences suggest that the protocols had comparable efficiency across castes. Among the reads mapped to the genome, more than 2.2 million positions included 2 alleles and of these, approximately 1.4 million positions exhibited allelic variation that enabled testing for associations with scouting. Whole genome analysis identified 137 variants associated (p-value < 0.0001) with scouting behavior. Functional categories corresponding to the genes linked to scouting variants involving neuronal, exoskeleton and immune processes offer insights into the molecular basis of the disposition of scouts to explore habitats and sharing information on food sources or of disposition of recruits to effectively provide food to the colony.

The detection of variants corresponding to ncRNA and genes with limited annotation suggests the opportunity of using a combination of genomic, transcriptomic and epigenomic tools to understand the molecular mechanisms underlying scout and recruit behaviors. Additional association studies of promising regions in LG10, LG5, and LG12 will advance the understanding of potentially causative variants.

## Supporting Information

S1 Table Summary of sequence read mapping to the *A*. *mellifera* genome among scouts and recruits. (CSV)

S2 Table Location, frequency, effect, and predicted ConSite transcription factors of all variants significant for scouting behavior. (CSV)
